# Converging seasonal prevalence dynamics in experimental epidemics

**DOI:** 10.1186/1472-6785-11-14

**Published:** 2011-05-17

**Authors:** Sandra Lass, Jürgen W Hottinger, Thomas Fabbro, Dieter Ebert

**Affiliations:** 1 Départment de Biology, Ecologie et Evolution, Université de Fribourg, Chemin du Musée 10, Fribourg, 1700, Switzerland; 2 Zoological Institute, Evolutionary Biology, University of Basel, Vesalgasse 1, Basel, 4051, Switzerland; 3 Department of General Pathology and Pathological Anatomy, Institute of Pathology, University Medical Centre, Albert-Ludwigs-University Freiburg, Breisacher Strasse 115a, Freiburg, 79106, Germany; 4 Study Coordination Center, University Hospital Basel, Schanzenstrasse 55, Basel, 4031, Switzerland

**Keywords:** *Daphnia magna*, microparasite, microsporidium, *Hamiltosporidium tvärminnensis*, *Octosporea bayeri*, population density

## Abstract

**Background:**

Regular seasonal changes in prevalence of infectious diseases are often observed in nature, but the mechanisms are rarely understood. Empirical tests aiming at a better understanding of seasonal prevalence patterns are not feasible for most diseases and thus are widely lacking. Here, we set out to study experimentally the seasonal prevalence in an aquatic host-parasite system. The microsporidian parasite *Hamiltosporidium tvärminnensis *exhibits pronounced seasonality in natural rock pool populations of its host, *Daphnia magna *with a regular increase of prevalence during summer and a decrease during winter. An earlier study was, however, unable to test if different starting conditions (initial prevalence) influence the dynamics of the disease in the long term. Here, we aim at testing how the starting prevalence affects the regular prevalence changes over a 4-year period in experimental populations.

**Results:**

In an outdoor experiment, populations were set up to include the extremes of the prevalence spectrum observed in natural populations: 5% initial prevalence mimicking a newly invading parasite, 100% mimicking a rock pool population founded by infected hosts only, and 50% prevalence which is commonly observed in natural populations in spring. The parasite exhibited similar prevalence changes in all treatments, but seasonal patterns in the 100% treatment differed significantly from those in the 5% and 50% treatments. Populations started with 5% and 50% prevalence exhibited strong and regular seasonality already in the first year. In contrast, the amplitude of changes in the 100% treatment was low throughout the experiment demonstrating the long-lasting effect of initial conditions on prevalence dynamics.

**Conclusions:**

Our study shows that the time needed to approach the seasonal changes in prevalence depends strongly on the initial prevalence. Because individual *D. magna *populations in this rock pool metapopulation are mostly short lived, only few populations might ever reach a point where the initial conditions are not visible anymore.

## Background

Seasonal changes in prevalence are ubiquitous in infectious diseases of plants, invertebrates and vertebrates [1-7]. Some of the more famous ones are influenza and measles, two of the paradigms for regular seasonal epidemics. Understanding the dynamics of such prevalence patterns has been a major challenge in epidemiology for the last decades. Numerous factors are known to cause and influence seasonal prevalence dynamics. Extrinsic factors affecting prevalence dynamics include physical conditions such as weather as well as community context of the host-parasite system, i.e. interactions with other members of the community [6-13]. Transmission of the influenza virus has been shown to depend on temperature and humidity and to be favoured by dry and cold conditions [14]. Intrinsic factors, inherent properties of the host-parasite system itself, include the host's immune system, host genetics, the parasite's transmission mode and parasite virulence, all of which are capable of generating seasonal changes in prevalence [15]. Seasonal prevalence patterns observed in natural populations may thus result from the complex interplay of different mechanisms. In measles, an external forcing (alternation of holidays and school terms) stimulates an intrinsic oscillatory behaviour of the host-parasite system caused by its short duration of infection and long-lasting host immunity [e.g. [16,17]].

Furthermore, seasonal prevalence patterns can have very different characteristics, even in the same system. For example, it is possible for multiple stable patterns to co-exist in one system [18]. Which pattern is approached by a system may again depend on multiple factors. Seasonal prevalence patterns could also be transient, with stable prevalence being reached if time permits [19]. The complexity of the interactions among factors influencing disease dynamics is hardly ever understood, even more so, as most studies of seasonality of prevalence, are observational and comparative [1,20] whereas empirical testing with experiments or manipulation are often impossible and hence rare. An ultimate test to understand the robustness of prevalence patterns is to perturb them in an experimental setting and discern the influence of individual factors. This is the central aim of the current study.

*Hamiltosporidium tvärminnensis *(formerly misidentified as *Octosporea bayeri *[21]) is a common parasite in natural rock pool populations of *Daphnia magna*. It undergoes seasonal epidemics, where prevalence rises in spring and decreases across winter [22]. The observed disease pattern suggests the existence of an annual pattern that is approached by all populations. In these natural populations seasonal prevalence changes seemed independent of the time since the parasite appeared for the first time in a given population: populations that had recently gotten infected showed the same pattern as populations that were known to be infected for years [22]. Experimental manipulations of the disease dynamics were however not conducted, leaving several questions about the nature and the stability of these dynamics open.

One major factor of interest for the *Daphnia - Hamiltosporidium *system is the role of the initial prevalence for the disease dynamics. The host species forms a metapopulation in rock pools, where individual populations undergo frequent extinction and recolonization [23]. This can lead to drastic differences in the initial prevalence of the parasites. In cases where the parasite invades an existing host population via spores or an infected resting egg the initial prevalence is rather low. In contrast, initial prevalence can be as high as 100%. This is because host populations are often founded by one or few resting eggs [24]. If all founder eggs are infected the initial prevalence is 100%, with lower values being possible for different proportions of founders being infected. Such widely differing initial prevalences are expected to strongly influence the disease dynamics in the short term, and possibly even in the longer term. Here we present a study to test the effect of initial prevalence of the disease dynamics of *Hamiltosporidium tvärminnensis*.

To investigate if initial prevalence may lead to different prevalence patterns, we conducted an epidemiological experiment starting with mesocosm populations differing widely in their initial prevalence (0, 5, 50 and 100%). The host-parasite system was isolated from its natural context, excluding other parasites and competitors that could potentially influence prevalence [9]. Other factors known to affect prevalence patterns, such as host and parasite genotype and host population density, were excluded. Experimental populations were started with one host genotype, the same mixture of parasite genotypes and the same density. Based on our observations in natural populations [22], we hypothesize that seasonal prevalence patterns are robust to initial prevalence differences in this system.

We further tested if the parasite affects host population density. Based on experiments with other *Daphnia *microparasites [25,26], we predict a parasite-induced reduction of host population density. The extent of parasite-induced reduction of host population density is expected to correlate with initial parasite prevalence. As the parasite is known to reduce host fecundity, and lower fecundity has been shown to result in lower host population density [25,26], we expect a stronger reduction in host density in populations with 100% initial prevalence compared to populations started with lower prevalence. If the parasite also reduces sexual reproduction in its host, this is likely to result in a lower host population density already in spring after the sexually-produced resting eggs hatched. If a lower hatchling density is observed in spring this could alternatively be explained by a lower hatching success of infected resting eggs which we will also test for.

### The host-parasite system

The host *Daphnia magna *is a planktonic freshwater crustacean that reproduces by cyclical parthenogenesis with clonal reproduction during favourable conditions and a switch to sexual reproduction when environmental conditions deteriorate. Sexual reproduction results in the formation of resting eggs enclosed by a so-called ephippial case (part of the mother's carapace), in which eggs can outlast winter frost and summer drought. When environmental conditions become favourable females hatch from these resting eggs and start clonal reproduction.

The parasite *Hamiltosporidium tvärminnensis *is a microsporidium specific to *D. magna *causing chronic infections. It is an obligate intracellular parasite infecting the fat cells and the ovaries of its host [27]. The parasite significantly lowers reproductive success, i.e. production of asexual offspring as well as of sexually produced resting eggs, and decreases longevity in its *D. magna *host as compared to uninfected hosts [28]. It has a direct life-cycle and transmits horizontally, via spores from dead decaying hosts and vertically, from mothers to their offspring [27]. Vertical transmission to asexual eggs is 100% efficient whereas transmission to sexual eggs is less efficient [22,27]. *Hamiltosporidium *is the most abundant microparasite in *D. magna *populations in the Tvärminne archipelago along the south-western coast of Finland [23] where host and parasite inhabit rainwater-filled depressions in the rocks of the Skerry islands. At least seven other ecto- and eight different endoparasites parasitize *D. magna *in these rock pools; and two other *Daphnia *species, *D. pulex *and *D. longispina*, as well as a number of freshwater invertebrates, including some which prey on *Daphnia*, co-occur with *D. magna *[23,29,30]. The role of these *Daphnia *antagonists for the dynamics of *Hamiltosporidium *is unknown. In the current experiment we excluded them, by conducting the experiment in mesocosms spatially isolated from the rock pool populations.

In the rock pools, *D. magna *hatch from resting eggs in spring (May), reproduce asexually (about 8 asexual generations per year) and sexually from spring to autumn (May-October) and undergo diapause during winter (November-April). The rock pools are characterized by instability caused by frequent drying up in summer, sudden invasions of brackish water from the Baltic Sea and freezing in winter. Summer drought of pools also leads to *Daphnia *diapause. Our experiment included winter diapause, but we excluded summer droughts and the invasion of brackish water.

Most importantly for our study, the parasite exhibits pronounced seasonal prevalence dynamics in natural populations [22]: Prevalence is low to intermediate in spring, increases rapidly across the summer, reaching close to 100% prevalence in most populations by mid summer. The following spring, prevalence is again at low to intermediate levels [22]. Prevalence increases due to a combination of perfect vertical transmission from mothers into their asexually derived offspring and efficient horizontal transmission enabling parasite spread [22,27]. The main cause identified for prevalence decrease is host diapause [22,31]. Several factors seem to contribute to this diapause-related prevalence decline: Vertical transmission into sexual resting eggs is imperfect, reducing prevalence after diapause [27]. Imperfect vertical transmission to sexual offspring is stronger in outbred sexual eggs than in selfed sexual eggs [31], making host genetic diversity a confounding factor in disease dynamics [32]. In our experiment, we therefore excluded the effect of host genetic diversity. Environmental conditions during diapause further influence the magnitude of the prevalence decrease [22]. Again, we excluded this factor in our experiment, by treating all populations equally during diapause.

This host-parasite system offers the unique opportunity to investigate seasonal prevalence patterns and to improve our understanding of regular prevalence dynamics using epidemiological experiments.

## Results

### Parasite prevalence

**Figure 1:**
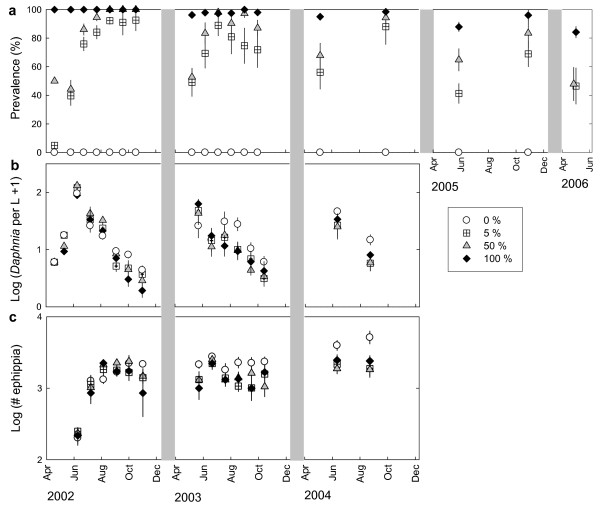
**Parasite prevalence, host population density and number of host resting eggs in the epidemiological experiment**. Means of parasite prevalence (a), of host population density (b) and of number of *Daphnia *ephippia (c) and their standard errors in experimental populations of the host *Daphnia magna *either uninfected or infected with the microsporidium *Hamiltosporidium tvärminnensis*. Host populations differed in their initial parasite prevalence in April 2002 (0%: n = 14, 5%: n = 10, 50%: n = 10 or 100%: n = 10), were kept outdoors and sampled regularly between 2002 and 2006.

**Figure 2:**
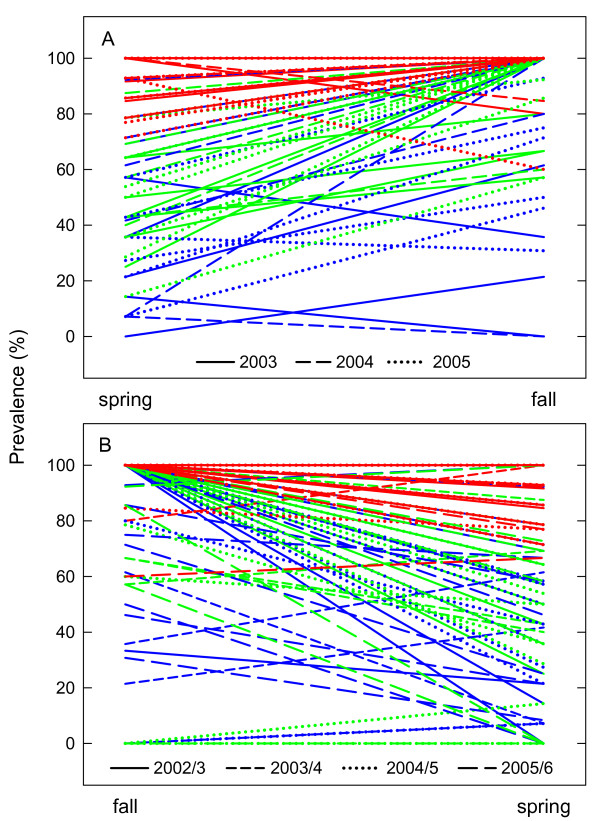
**Parasite prevalence in the epidemiological experiment**. (A) Increase of parasite prevalence across summer and (B) Decrease of prevalence across winter analysed to test for differences in seasonal disease patterns between experimental host populations of the host *Daphnia magna *infected with the microsporidium *Hamiltosporidium tvärminnensis*. Host populations differed in their initial parasite prevalence in April 2002 (5%: n = 10, blue lines, 50%: n = 10, green lines or 100%: n = 10, red lines). Different line types represent different years. Missing lines result from prevalence data missing for individual populations.

Parasite prevalence increased significantly during the hosts' growing season from spring to autumn (LMM; 28% increase, t = 10.61, p < 0.001) and decreased significantly across winter diapause, i.e. from fall to spring (LMM; 23% decrease, t = -8.14, p < 0.001, Figures 1a and 2). Prevalence changes in the 50% and 100% treatments differed significantly from each other (LMM; difference in increase and decrease, t > 5.26, p < 0.001, for both contrasts) while we did not find any evidence for a difference between the 5% and 50% treatment (LMM; t < 0.87, p > 0.35). The midsummer prevalence decline in the 5% and 50% treatments in 2003 (Figure 1a) was caused by the fact that the planktonic *Daphnia *population went extinct in a few populations, and prevalence was low in two of three populations after they got repopulated by hatchlings from resting eggs.

### Host population density

**Figure 3:**
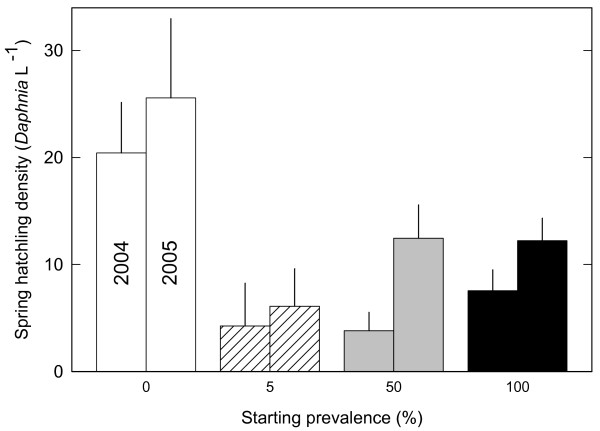
**Density of host hatchlings in spring in the epidemiological experiment**. Mean spring density and standard errors of *Daphnia magna *hatchlings in experimental populations either uninfected or infected with the microsporidium *Hamiltosporidium tvärminnensis*. Host populations differed in the initial parasite prevalence in April 2002 (0%: n = 14, 5%: n = 10, 50%: n = 10 or 100%: n = 10) and were kept under outdoor conditions. Hatchling densities were estimated in 2004 and 2005 after hatchlings had been detected in all populations.

Host density in uninfected populations (0% treatment) was on average about 1/3 higher than host density in infected populations (LMM; t = 2.83, p = 0.007, Figure 1b). Host density of the infected treatments did not differ from each other (LMM; t < 0.06, p > 0.9, Figure 1b). Spring hatchling density differed significantly between treatments (one-way ANOVAs, 2004: F_3, 34 _= 3.73, p = 0.02; 2005: F_3, 37 _= 4.89, p = 0.006), with uninfected populations harbouring more hatchlings than infected populations (Figure 3). Infected populations did not differ significantly in hatchling density.

### Number of ephippia and resting eggs

Time had a significant effect on the number of ephippia produced (repeated-measures ANOVA, time: F_13, 27 _= 17.86, p < 0.001). With increasing time the treatments differed in the number of ephippia they harboured (repeated-measures ANOVA, treatment × time: F_39, 81 _= 2.17, p = 0.0018) with a higher number of ephippia accumulating in uninfected than in infected populations (Figure 1c). Treatment alone did not have a significant effect on the number of ephippia (repeated-measures ANOVA, treatment: F_3, 39 _= 2.12, p = 0.114). As in natural rock pools water volume in the experimental containers was highly variable (between 27-100 L) over time. This was due to rain, evaporation and sampling. However, water level fluctuations went in parallel in all populations and thus did not affect treatments differently.

Ephippia from uninfected populations contained on average about 18% more resting eggs (1.47 ± 0.04 eggs per ephippium) than those from infected populations (5%: 1.15 ± 0.08, 50%: 1.18 ± 0.10, 100%: 1.31 ± 0.07; one-way ANOVA, F_3,23 _= 4.19, p = 0.019; sample size n = 49 ± 3 (s.e.) ephippia per population).

### Hatching success

After winter diapause, hatchlings appeared in all experimental populations around the same time in spring (S. Lass, personal observation). Comparing percentage of loculi in the ephippia filled with resting eggs before and after winter diapause showed that on average 44.5% ± 0.4 (s.e.) of all eggs produced in 2002 hatched in spring 2003 (with 44.7% ± 4.4 in the uninfected controls, 45.6% ± 4.1 in the 5%, 43.1% ± 7.0 in the 50% and 45.1% ± 6.6 in the 100% treatments). Consequently, hatching success did not differ significantly between treatments (one-way ANOVA, F_3, 23 _= 0.018, p = 0.99).

## Discussion

### Seasonal Prevalence pattern

Disease dynamics of the microsporidium *Hamiltosporidium tvärminnensis *in populations of its *Daphnia magna *host are characterized by pronounced seasonality: Prevalence is low in spring and high in fall in natural [22] as well as in experimental populations (Figure 1a). These prevalence dynamics were found in populations which were started with very different initial prevalence (Figure 1a). Although populations started with 100% prevalence exhibited a decrease in prevalence after the first winter (to 96 ± 2% prevalence) and a regular seasonal increase and decrease in prevalence thereafter, prevalence changes in these populations differed significantly from those of the 50% treatment due to their lower amplitude (Figures 1a, 2).

Prevalence in the 100% treatment can be explained by the following: During the first summer the 100% efficient vertical transmission to asexually-produced host eggs [22,27,31] kept prevalence at 100%. The decrease in prevalence in this treatment after the first winter was likely caused by a combination of imperfect vertical transmission into sexual eggs [27,31] and higher mortality of infected compared to uninfected resting eggs during winter diapause [22]. The slow onset of prevalence dynamics in the 100% treatment may further be explained by the accumulation of parasite spores. The high prevalence in this treatment, leads to a large pool of infective *H. tvärminnensis *spores early on, acting like a spore bank. These spores can survive several months to a year outside their host (S. Lass & D. Ebert, personal observation) and thus may accumulate to temporarily high levels. In particular, high prevalence early in the season, when high *Daphnia *densities lead to many dead infected hosts, can contribute to this effect. Given enough time it is possible that the dynamics of the 100% treatment may eventually become indistinguishable from those of the other treatments. In our experiment, four years were not enough to answer this question. Given that the life span of most natural rock pool *Daphnia *populations is very limited [33], it is unlikely that the seasonal disease patterns will have a chance to persist for long enough to become indistinguishable in populations differing strongly in initial prevalence under conditions as in our experiment. While we excluded host migration in our experiment, migration of *Daphnia *is common in natural populations [33,34]. Uninfected immigrating *Daphnia *experience a fitness benefit when invading a rockpool harbouring an infected *Daphnia *population, and this advantage becomes even larger with increasing parasite prevalence in the resident population [35]. Thus, immigration of *Daphnia *resting eggs is likely to affect prevalence patterns in natural populations and could contribute to synchronization of prevalence dynamics in populations with different initial prevalence.

The seasonal dynamics of *Hamiltosporidium *have two components: prevalence increase over summer and prevalence reduction after host diapause. The increase in prevalence over summer is caused by horizontal transmission of the parasite via spores from dead infected to uninfected hosts [22]. This is a one-way road, as an infected line cannot loose the parasite anymore as long as it reproduces asexually. Declines in prevalence during the summer are the consequences of low vertical transmission efficiency and higher reproductive success of uninfected compared to infected females [31,36], which may occur under low densities and good food condition.

Several mechanisms contribute to the prevalence decrease associated to host diapause: First, the onset of resting egg production early in the year when prevalence is still low (Figure 1c) results in uninfected resting eggs produced by uninfected females. This is consistent with data on natural populations, showing that ephippia production peaks in mid summer [37]. This effect is enhanced by infected females producing a higher proportion of male offspring [38] which do not transmit the parasite and leaving more ephippia being produced by the uninfected females. Second, infected females have a lower fecundity than uninfected females [38] which is further manifested by our finding that ephippia from infected populations contained about 18% fewer resting eggs than those from uninfected populations. The difference in fecundity gains relative importance when infected hosts compete with uninfected hosts [36]. Thus, we speculate that a larger number of uninfected resting eggs is produced in populations containing infected and uninfected hosts than suggested by the proportion of uninfected hosts at a given time. Third, infected ephippia have a higher mortality during diapause than uninfected ephippia [22]. Taken together, hatching of resting eggs in the following spring results in prevalence which is lower than that observed in the previous fall, i.e. before diapause. Our study rules out a few factors that could have contributed to the lower prevalence in spring: First, we found no evidence for differences in the time of hatching between uninfected and infected resting eggs. Hatchlings appeared simultaneously in all populations in spring and thus, hatching of uninfected and infected resting eggs was synchronized. Second, we did not find any difference in hatching success between infected and uninfected populations. We speculate that also within populations, hatching success of infected and uninfected resting eggs did not differ. Third, prevalence changes are unlikely to be caused by immigration from other populations. We have no evidence for migration as uninfected populations remained uninfected throughout the whole experiment and no *Daphnia *ever occurred in the two additional *Daphnia *free containers.

Our experiment was set up to exclude a number of confounding factors which were suggested to play a role for disease dynamics under natural conditions. First, host genetics have been shown to play an important role in host susceptibility and disease persistence in *Daphnia *[39-41], and epidemics have been suggested to be terminated by rapid changes in genotype composition of host populations [42]. We show that in the near absence of genetic variation, parasite prevalence still exhibited a regular increase and decrease, excluding host genetic heterogeneity as a necessary factor for this pattern. Our experimental populations consisted of a single susceptible host clone during the first growing season, which diversified after the first winter when hosts hatched from eggs produced by selfing. Such a low genetic diversity is not untypical for the natural conditions in the metapopulations in southern Finland, where new populations are frequently founded by single *Daphnia *clones [24].

Second, predators, competitors and other parasites can strongly influence host-parasite interactions and may considerably alter disease dynamics [8,10,43]. Our previous field observations could not rule out that prevalence dynamics may have been caused by the interplay of multiple factors and players rather than by the interaction between one host and one parasite species. Here, we isolated the host-parasite system from its natural community context and still observed the same seasonal prevalence pattern. The few potential *Daphnia *predators and aquatic insect larvae that invaded temporarily into some of the mesocosms were rare and different from those characteristic to the Finnish rock pool communities [29,30]. We conclude that other community members thus do not substantially contribute to the regular prevalence dynamics in this host-parasite system.

### Parasite-mediated host density reduction

Infection with *H. tvärminnensis *reduced the density in populations of its host *D. magna*. Different initial prevalence, however, was not reflected in the extent of density reduction. Comparing host population densities during three years, we found that density in uninfected host populations on average exceeded that of infected populations by one third (Figure 1b). This is consistent with theoretical [44-47], observational [2] and experimental [25,26,48-50] studies showing that parasites that affect host fecundity can affect host population density. Most former studies demonstrating microparasite-driven reduction of host density in *Daphnia *have been conducted under constant conditions in the laboratory, most notably under regular food supply [25,26]. Here, we demonstrate that an effect of parasitism on host density is even detectable under outdoor conditions that include fluctuating food supply, varying temperature and changing water volume. A recent long-term study has also been able to demonstrate effects of parasite infection on host population density in natural *Daphnia *populations [6].

From the second year onwards infected populations exhibited lower spring hatchling density (Figure 3) compared to uninfected populations. Several mechanisms contributed to lower spring hatchling densities. First, lower hatchling densities in 2004 and 2005 result from the lower number of ephippia produced in infected populations in previous years (Figure 1c). Population density and number of ephippia are likely to reinforce each other as populations of lower density are likely to produce fewer ephippia leading to lower hatchling density. Second, we found that ephippia in infected populations include fewer resting eggs than ephippia in uninfected populations. Third, in a previous study we found that infected resting eggs have lower survival probability in winter than uninfected resting eggs [22].

Lower hatchling densities in spring and reduced densities throughout the summer may have important consequences for the long term survival of infected *D. magna *populations in the metapopulation our hosts and parasites originated from [23]. In this metapopulation *D. magna *frequently goes locally extinct [33]. The causes for this extinction in these rock pools are often linked to abiotic factors, such as summer droughts or flooding by sea water. Populations may survive such catastrophic events when sufficient numbers of viable resting eggs are present. Our finding, that hatchling densities in infected populations is reduced, suggests that the parasite may contribute to population extinction in this metapopulation. Long term data on the metapopulation dynamics and the distribution of the parasite are needed to test this hypothesis.

## Conclusions

Here, we show that the combination of well-controlled experiments and knowledge of the biology of the host-parasite system under study allows understanding seasonal prevalence patterns. In addition to long-term observations of disease dynamics in natural populations and mathematical models [6,7,12,22], experimental epidemiology is a powerful tool contributing significantly to our understanding of the causes, mechanisms and consequences of disease dynamics [48,51,52].

## Methods

### Epidemiological experiment

We used a single *D. magna *clone that was the product of out-crossing between two uninfected clones from two rock-pool populations (4 km apart from each other) in southern Finland. The clone was kept in the lab for four years prior to the experiment. We used a mixture of seven different *H. tvärminnensis *isolates from the Finnish rockpools to infect the host clone [53]. Infected and uninfected *Daphnia *were cultured in the same way. On April 18, 2002, the experiment was started by transferring *D. magna *of different age into 100-L plastic containers, which were filled with 48 L artificial medium [modified after [54], without adding any well water, [55]]. Infected and uninfected *Daphnia *were mixed to obtain 14 populations without any infected *D. magna *(0% prevalence), ten populations with 5%, ten with 50% and ten with 100% starting prevalence. All infected populations contained the same mixture of parasite isolates. Under natural conditions a low number of hosts would found a population. However, as we were interested in the effect of parasite prevalence and did not want to mingle potential effects of host population bottlenecks and parasite effects on the host population, we started all populations with the same number of hosts, 240 *D. magna *(resulting in 12 infected *D. magna *in the 5% treatment, 120 in the 50% and 240 in the 100% initial prevalence treatment). To avoid starvation in the newly set up containers, we fed all populations four times with 1 × 10^9 ^cells of the green algae *Scenedesmus obliquus *during the first two weeks of the experiment. Thereafter, food was not added again.

Containers were placed on a plain meadow in the Botanical Garden of the University of Fribourg, Switzerland. They were arranged in a grid with equal distances (1 m) between the containers. Every October, the containers were covered with nets (meshsize 7 mm) to prevent leaves from falling into the water, which would have made sampling difficult. In November, water levels were lowered to 24 L to avoid bursting of containers due to ice, and containers were closed with a lid and covered with a black, photo resist plastic foil to produce complete darkness in all containers. Air flow was possible under the foil to prevent anoxic conditions. Every spring (in March or April), the foil and lids were removed and 24 L artificial medium were added to each container. We had two additional containers without *Daphnia *within the grid to measure the change in water volume caused by evaporation, rain and sampling. The mean volume of these two containers served to get an estimate for water volume of all other containers. This was used to estimate the total number of ephippia present in the different populations (see below). Uninfected populations as well as the additional containers were also used to detect if the parasite and/or the host migrated between containers.

### Sampling

After the foil had been removed each spring, populations were checked daily until the first *Daphnia *hatchlings occurred. From April to November, populations were sampled every other week in 2002 and 2003, every 5 weeks in 2004, three times in 2005 and once in 2006. In 2006, we omitted sampling the uninfected populations. From 2002-2004, we alternately estimated parasite prevalence and host population density. We mixed the populations well prior to sampling a certain water volume from the populations. Thus, sampling altered host population size but did not change population density. Four litres (8 samples of 500 mL each) were taken from each population with exception of the density samples from May 8, Jul 3, 2002, and Jul 16, 2003 when only 2 L were sampled because of low water levels. Each sample contained the organisms present at that time (microorganisms, algae, *Daphnia*, other aquatic invertebrates that had invaded the containers), ephippia and debris. All treatments were always sampled in the same way.

Besides *D. magna*, we never found any other *Daphnia *species in our mesocosms. No other endo- or ectoparasites of *Daphnia *were observed except for one occasion when we found an unknown microsporidian parasite in a single *Daphnia*. As this parasite grouped with a clade of microsporidia of mosquitoes in a molecular phylogeny [56], it may have been an accidental infection originating from an immigrating mosquito. Besides, Diptera larvae (Chironomidae and Culicidae; in 35-44 of the 44 mesocosms), other aquatic invertebrates were rare: Baetidae larvae (mayflies) in maximal 3 of 44 mesocosms at a time, occasionally a water strider (Gerridae), one *Notonecta *specimen (backswimmers) once and on two occasions one specimen of Dytiscidae (water beetles). The commonness of the Diptera larvae and the rareness of other insects in our mesocosms make it unlikely that our treatments are significantly influenced by them.

### Parasite prevalence

Infection with *H. tvärminnensis *can be assessed via microscopic detection (phase contrast, 400 × magnification) of the typical spores about 8-12 days after the start of an infection or, in case of vertical transmission, at an age of about 8 days [57]. Therefore, we kept *Daphnia *sampled for prevalence estimates individually in 100-mL jars under laboratory conditions (artificial medium, regular food supply, 20°C) for 10-14 days to ensure sufficient spore development. Fourteen animals from each infected population were randomly chosen. If there were less, we took all the ones present in a sample. Wet-mount preparations of all *Daphnia *were microscopically checked for *H. tvärminnensis *presence and the presence of other parasites.

To check if the uninfected controls became contaminated by the parasite and to maximize the detection of even low prevalence, 50-100 *Daphnia *(if possible, otherwise all that we sampled) from each uninfected population were kept in 1 L medium for two weeks to allow infections by possible parasites to develop. After this incubation time, *Daphnia *were homogenized and the suspension checked for parasite spores. This method would allow finding a single infected host among 100 uninfected.

For the infected populations, we tested if prevalence increased significantly across host growing season and if it decreased across host winter diapause. Prior to analysis, we calculated the prevalence changes across seasons: We took the difference between the first prevalence estimate in spring and the last in fall of the same year and the difference between the last prevalence estimate in fall and the first in spring of the following year. Because the prevalence at the start of the experiment was imposed by the treatment (and had an error variance of zero), prevalence data from spring 2002 were omitted in this analysis. Data were analyzed with linear mixed effect models (LMM) accounting for heteroscedasticity among treatments [58]. Sequential contrasts between the treatment levels 5% and 50%, as well as 50% and 100% were used to estimate and test the difference in prevalence increase or decrease.

### Host population density

The contents of 2 L sampled from each population were filtered (mesh size 250 μm) and preserved in 96% ethanol prior to counting. *Daphnia *and free ephippia were counted using a stereomicroscope (10-20 × magnification) whereby we also recorded the presence of other aquatic animals in the sample, if present. In order to test if *Daphnia *population density depends on the treatment, we analyzed the data with a linear mixed effect model (LMM) after *log(x *+ 1) transformation [58]. We excluded the two initial densities in 2002 from the analysis as all populations were started with the same density. Sequential contrasts between the treatment levels 0% and the mean of 5%, 50% and 100%, as well as 5% and 50% and between 50% and 100% were used to estimate and test the difference in *Daphnia *density. The statistical analysis took the repeated measurements from the same populations into account, but ignored the order of samplings.

In order to test for differences in population densities between treatments in spring, we estimated the density of hatchlings. We took further samples in spring 2004 and 2005, after *Daphnia *hatchlings had appeared in all populations. From each population 2 L were sampled and *Daphnia *were immediately counted on site. After counting, the hatchlings were reintroduced into their population of origin to minimize disturbance. Hatchling densities were analyzed separately from the other host population densities. To test for differences in spring hatchling density (*log*-transformed), we carried out two one-way ANOVAs with treatment as fixed factor.

### Number of ephippia and resting eggs

As *H. tvärminnensis *lowers reproductive success in its host, the number of ephippia was expected to differ between prevalence treatments. To test for such differences, ephippia were counted in the well-mixed density sample (see above). This count was multiplied with the ratio of water volume in the experimental population/sample volume to obtain an estimate of the total number of ephippia for each population at that time. We report ephippia estimates based on these samples without correction for the loss of ephippia in the previous samples. Therefore total ephippia production is under estimated, but to the same degree in all replicates and thus numbers can be compared between treatments. Due to the very dry summer 2003, water volumes were lower than in other years, which lead to a stronger sampling effect and thus stronger underestimation of total ephippia production. The number of ephippia (*log*-transformed) was analyzed by repeated-measures ANOVA with treatment as fixed factor.

### Hatching success

It was not known if the presence of *H. tvärminnensis *infection affects hatching of resting eggs. Such a differential hatching success could result in differences in hatchling and population density. To test for potential differences in hatching success in different treatments, we estimated the proportion of resting eggs that hatched after the first winter diapause (2002/03). We opened ephippia taken from six populations of each treatment in spring 2003 before the onset of sexual reproduction (i.e. before *Daphnia *with ephippia were observed), counted the resting eggs they contained and calculated the proportion of filled loculi. Each ephippium can contain two resting eggs, i.e. has two loculi. The proportion hatched (*arcsine*-transformed) was estimated as the difference between the percentages of loculi filled before and after winter diapause and analyzed by one-way ANOVA with treatment as fixed factor.

For all statistical analyses, we used either R including package nlme [58] or Jmp In 5.1 [59]. Model assumptions were carefully inspected using several different diagnostic plots.

## Authors' contributions

SL planned and carried out the experiments, analysed the data and wrote the manuscript. JWH contributed to planning and carrying out the experiments. TF helped with the analysis of the data and the interpretation of the results. DE contributed to the planning of the study and to revising the manuscript. All authors approve the publication of the study.
